# Medicaid Coverage for Tobacco Dependence Treatments in Massachusetts and Associated Decreases in Smoking Prevalence

**DOI:** 10.1371/journal.pone.0009770

**Published:** 2010-03-18

**Authors:** Thomas Land, Donna Warner, Mark Paskowsky, Ayesha Cammaerts, LeAnn Wetherell, Rachel Kaufmann, Lei Zhang, Ann Malarcher, Terry Pechacek, Lois Keithly

**Affiliations:** 1 Massachusetts Tobacco Control Program, Boston, Massachusetts, United States of America; 2 Office of Medicaid Commonwealth of Massachusetts, Boston, Massachusetts, United States of America; 3 Office on Smoking and Health, National Center for Chronic Disease Prevention and Health Promotion, Centers for Disease Control and Prevention, Atlanta, Georgia, United States of America; 4 Massachusetts Tobacco Control Program, Boston, Massachusetts, United States of America; Mount Sinai School of Medicine, United States of America

## Abstract

**Background:**

Approximately 50% of smokers die prematurely from tobacco-related diseases. In July 2006, the Massachusetts health care reform law mandated tobacco cessation coverage for the Massachusetts Medicaid population. The new benefit included behavioral counseling and all medications approved for tobacco cessation treatment by the U.S. Food and Drug Administration (FDA). Between July 1, 2006 and December 31, 2008, a total of 70,140 unique Massachusetts Medicaid subscribers used the newly available benefit, which is approximately 37% of all Massachusetts Medicaid smokers. Given the high utilization rate, the objective of this study is to determine if smoking prevalence decreased significantly after the initiation of tobacco cessation coverage.

**Methods and Findings:**

Smoking prevalence was evaluated pre- to post-benefit using 1999 through 2008 data from the Massachusetts Behavioral Risk Factor Survey (BRFSS). The crude smoking rate decreased from 38.3% (95% C.I. 33.6%–42.9%) in the pre-benefit period compared to 28.3% (95% C.I.: 24.0%–32.7%) in the post-benefit period, representing a decline of 26 percent. A demographically adjusted smoking rate showed a similar decrease in the post-benefit period. Trend analyses reflected prevalence decreases that accrued over time. Specifically, a joinpoint analysis of smoking prevalence among Massachusetts Medicaid benefit-eligible members (age 18–64) from 1999 through 2008 found a decreasing trend that was coincident with the implementation of the benefit. Finally, a logistic regression that controlled for demographic factors also showed that the trend in smoking decreased significantly from July 1, 2006 to December 31, 2008.

**Conclusion:**

These findings suggest that a tobacco cessation benefit that includes coverage for medications and behavioral treatments, has few barriers to access, and involves broad promotion can significantly reduce smoking prevalence.

## Introduction

Cigarette smoking continues to be the leading cause of preventable morbidity and mortality in the United States [1]. Despite recent overall declines in smoking prevalence in the United States, the prevalence in the Medicaid population – the health insurance program for the poor - has remained 65% higher than in the rest of the population [2], [3]. In Massachusetts alone, smoking causes $1 billion annually in excess health care costs to the Medicaid program. In April 2006, the Massachusetts legislature passed Chapter 58 of the Acts of 2006 (“An Act Providing Access to Affordable, Quality, Accountable Health Care”) requiring all individuals in Massachusetts to have health insurance. In an effort to reduce smoking prevalence in the Medicaid population, the law mandated coverage for two types of tobacco cessation treatment: behavioral counseling and all Food and Drug Administration (FDA)-approved medications. Prior to 2006, MassHealth (the Massachusetts Medicaid program) did not provide tobacco cessation benefits.

With the implementation of this benefit, MassHealth subscribers are allowed two 90-day courses per year of FDA-approved medications for smoking cessation, including OTC medications like nicotine replacement therapy, and up to 16 individual or group counseling sessions. Medications require written prescriptions following an office visit. Prior authorization is not required to prescribe the nicotine patch, gum, lozenge, Chantix, or bupropion/Wellbutrin. With prior authorization, the nicotine inhaler and nasal spray may also be covered. The co-payment is minimal at $1.00 or $3.00. Detailed information on the benefit design and reimbursement rates is available at www.makesmokinghistory.org/quitworks/masshealth.html. A total of 70,140 unique MassHealth subscribers used the newly available benefit between July 1, 2006 and December 31, 2008, i.e., approximately 37% of all Medicaid smokers. All utilization data reported in this paper were obtained from MassHealth claims data.

It is the objective of this study to determine if smoking prevalence decreased in the Massachusetts Medicaid population after the initiation of mandated tobacco cessation coverage.

## Methods

### Data Source

The Behavioral Risk Factor Surveillance System (BRFSS) is the largest continuously conducted telephone health surveillance system of adults in the world [4]. The BRFSS is a state-based, cross-sectional telephone survey conducted by state health departments with technical and methodological assistance provided by the Centers for Disease Control and Prevention (CDC). BFRSS surveys focus data collection on behaviors, in contrast to attitudes or knowledge. States use BRFSS data to identify emerging health problems, establish health objectives, and track their progress toward meeting these objectives [4].

### Main Outcome Measures

Although the BRFSS covers a wide variety of questions about health behaviors, this work focuses on responses to questions about tobacco use. Smoking status is divided into three groups: current smokers, former smokers, and never smokers. Current smokers are defined as having smoked at least 100 cigarettes in their lifetimes and smoke currently. Former smokers are defined as having smoked at least 100 cigarettes in their lifetimes but did not smoke currently. Individuals who have not smoked at least 100 cigarettes in their lifetimes were classified as never smokers. Quit attempts were measured by counting individuals who had stopped smoking for 1 day or more during the preceding 12 months in an attempt to quit. Recent quits were counted using individuals who had stopped smoking within the previous 12 months.

### Other Variables of Interest

Unlike most states, the Massachusetts BFRSS includes health insurance questions. These questions make it possible to distinguish respondents by insurance status including coverage by MassHealth. Because the MassHealth tobacco cessation benefit was limited to subscribers between the ages of 18–64, this study was also limited to MassHealth subscribers ages 18–64. Approximately one in six (16%) Massachusetts BRFSS survey respondents identify their health insurance as MassHealth, the Massachusetts Medicaid program.

### Statistical Analysis

This study is fundamentally ecological in nature; therefore, it was not possible to link specific utilization behavior with individual quits or quit attempts. Consequently, this analysis will look at the available BRFSS data from three perspectives in order to provide greater confidence in the results reported. Tests of proportional differences were followed by a trend analysis which was followed by logistic regression.

At the most basic level, differences in proportions were evaluated using t-tests. Since health care reform legislation in Massachusetts expanded eligibility for the MassHealth program, difference estimates were computed for population samples that were adjusted for demographic changes in the post-benefit period. Demographically-adjusted rates were calculated in such a way that the demographic characteristics of the post-benefit period (July 1, 2006 – December 31, 2008) were forced to match those in a specified pre-benefit period (January 1, 2003 – June 2006). Adjustments were made for age, gender, education, and race/ethnicity. Age was grouped into 5 categories: 18–24, 25–34, 35–44, 45–54, and 55–64 year olds. Education status was classified as (1) less than a high school education, (2) high school graduate or GED, (3) 1 to 3 years of college, or (4) 4+ years of college. Race/ethnicity was categorized as (1) white, non-Hispanic or (2) other.

For all tests of proportional differences, the sample population used in the pre-benefit period included only MassHealth subscribers despite an increase in MassHealth enrollment post-benefit. By December 2008, the number of adults covered by MassHealth increased by 11.3% when compared to 2006 levels. A 2009 Kaiser Commission study estimated that 76,000 previously uninsured adults received coverage through MassHealth by December 2008 [5]. Since the increased enrollment in MassHealth was only slightly higher than the 76,000 estimated by Kaiser, it would be tempting to include all uninsured adults in any analysis of the pre-benefit period. However, the Kaiser report also estimates that more than four times as many previously uninsured adults (354,000) obtained insurance coverage through other programs. The majority obtained private health insurance or used the state's subsidized insurance program (Commonwealth Care). As a result, including uninsured adults in the pre-benefit population would likely overestimate the impact of the uninsured within the total MassHealth population.

In addition to tests of proportions involving smoking prevalence, quit attempts as well as the success of those attempts also were examined. It was hypothesized that a result indicating a decrease in smoking prevalence that was coincident with the implementation of the MassHealth tobacco cessation benefit could occur for two reasons. First, more smokers could be making quit attempts. Or second, more smokers could be making successful quit attempts.

Trend analyses were computed using joinpoint analysis. The National Cancer Institute publishes joinpoint analysis software as a tool for assessing public health trends. More information on joinpoint analysis is available at the National Cancer Institute website at http://srab.cancer.gov/joinpoint/. The joinpoint software takes trend data and fits the simplest joinpoint model that the data will allow. No minimum or maximum joinpoints were specified for the models used in this analysis, thereby allowing the joinpoint software to select the most appropriate model for the data. The basic data element of the joinpoint analysis was a demographically-adjusted smoking prevalence estimate. These were computed for each six month period between 1999 and 2008.

Compared to the analysis of differences in proportions described above, a longer time period was used for the joinpoint analysis. This decision was made because a shorter time period would have reduced the likelihood of finding multiple joinpoints in the pre-benefit period. It was considered important to know whether post-benefit smoking prevalence levels in the MassHealth population had been matched in some earlier period. A longer time period increased the likelihood of seeing trends that would contradict the hypothesis that decreased prevalence might be attributed to the initiation of cessation coverage.

Finally, a logistic regression was computed so as to make individual level adjustments for demographics. Here, the target variable was current smoking status as recorded by the BRFSS. In addition to demographic variables, two more variables were added to the logistic model to assess trends. First, a monthly sequential variable from January 1999 to December 2008 was included. This was used to measure long-terms trends in prevalence. Second, a monthly sequential variable beginning in July 2006 and ending December 2008 was also included to capture trends that were limited to the post-benefit period. The demographic variables used in the analysis were gender, race/ethnicity, education status, and age which could account for changes in demographics.

The BRFSS survey has a multistage probability sample design. BRFSS data are directly weighted for the probability of selection of a telephone number, the number of adults in a household, and the number of telephones in a household. A final post-stratification adjustment is made for non-response and non-coverage of households without telephones. The weights for each factor are multiplied together to get a final weight. All reported estimates were weighted using statistical analysis software (SAS version 9.1). The a priori significance level used for statistical tests was 0.05.

## Results

Smoking prevalence was examined in the MassHealth population from 1999 through 2008 using the Behavioral Risk Factor Surveillance System (BRFSS). The number of BRFSS respondents who were eligible for the benefit (i.e., MassHealth members aged 18 to 64) ranged from 402 respondents in 1999 to 1,969 respondents in 2008. Depending on the specific analysis, different time periods were studied.

It should be noted that the BRFSS relies on self-reports including questions about insurance status. To test the accuracy of self-reports about insurance, Massachusetts conducted a call-back survey with a subset of BRFSS respondents in 2007. The second call to the respondent took place an average of 31 days after the first call. The call-back survey found that more than 90% of respondents who had previously indicated that they had MassHealth coverage were able to confirm the presence of a valid MassHealth logo on their insurance card. The 2007 call-back survey suggests that the reliability of self-reported MassHealth subscriber status is high. (Source: Unpublished results of Massachusetts BRFSS 2007 follow-up interviews)

The unadjusted or crude estimate of smoking prevalence was significantly higher in the pre-benefit period when compared to the post-benefit period (p<.05). Specifically, smoking prevalence between 1/1/2003 and 6/30/2006 for MassHealth members 18–64 was estimated to be 38.3% (95% C.I. 33.6%–42.9%). In calendar year 2008, the most recently available data, prevalence for this population was estimated to be 28.3% (95% C.I.: 24.0%–32.7%). The above analyses were not adjusted for demographic changes in the post-benefit population.

Two comparisons were made with the demographically adjusted rates. First, the demographically adjusted rates were compared to the unadjusted rates for the post-benefit period. Here, there were no significant differences between the adjusted and unadjusted rates for current smoking prevalence, percentage of smoker making quit attempts, and recent quit success. See Table 1 for details. The absence of significant differences suggests that the effects of demographic changes in the MassHealth population with respect to smoking behavior were minimal.

**Table 1:** Comparison of Pre-, Post-Benefit Periods on Smoking Prevalence And Quitting Behavior.

	Pre-Benefit Period January 1, 2003 to June 30, 2006		Post-Benefit Period January 1, 2008 to December 31, 2008			
Measure	Crude%	95% C.I.	Crude %	95% C.I.	Dem-Adj %	95% C.I.
Current smoking	38.3	33.6 – 42.9	28.3*	24.0–32.7	28.8*	24.3 – 33.3
Quit Attempt	62.6	55.9 – 69.4	67.2	59.6 – 74.8	67.6	60.5 – 74.7
Recent Quit Success	6.6	3.8 – 9.3	18.9*	10.2 – 27.7	19.1*	13.0 – 25.2

*Statistically significant at the .05 level.

Data Source: BRFSS 2003 – 2008.

Given the above result, comparisons also were made for smoking behavior between the pre-benefit period and post-benefit period using the demographically adjusted rates. As shown in Table 1, there were significant differences in the rate of current smoking (38.3% pre-benefit vs. 28.8% post-benefit) and recent quit success (6.6% pre-benefit vs. 19.1% post-benefit) using the demographically adjusted data. There were no differences for the percentage of smokers making quit attempts. For a full breakdown of the demographics for the pre-benefit period compared to the post-benefit period, see Table 2.

**Table 2:** Demographics for the MassHealth Population, Age 18–64 for the Pre- and Post- Benefit Periods with Crude Smoking Rates.

	Pre-Benefit Period January 1, 2003 to June 30, 2006				Post-Benefit Period January 1, 2008 to December 31, 2008				
Characteristic	Sample Size	Weighted Sample Size	%	Crude Smoking (%)	Sample Size	Weighted Sample Size	%	Crude Smoking (%)	% Change in Smoking
Overall Population	2,016	892,919	100	38.3	1,969	454,851	100	28.3*	−26%
Gender									
Male	414	264,897	29.7	41.8	561	174,919	38.5*	28.4	−32%
Female	1,602	628,022	70.3	36.8	1,408	279,931	61.5*	28.3	−23%
Age									
18–24	295	265,878	29.8	38.1	214	132,809	29.2	22.6	−41%
25–34	550	241,466	27.0	42.9	377	97,685	21.5	33.8	−21%
35–44	530	212,151	23.8	35.7	427	80,245	17.6*	34.4	−4%
45–54	383	107,135	12.0	42.6	534	91,774	20.2*	31.0*	−27%
55–64	258	66,289	7.4	23.2	417	52,338	11.5*	18.5	−20%
Education status									
< HS	534	155,736	24.9	36.1	441	61,627	19.2	39.6	+10%
HS graduate	604	232,242	37.1	43.0	582	107,146	33.4	31.7	−26%
College 1–3 years	389	155,035	24.8	42.1	411	81,309	25.4	31.2	−26%
College 4+ years	192	82,618	13.2	22.9	312	70,632	22.0*	20.1	−12%
Race/ethnicity									
White, non-Hispanic	970	477,377	53.9	51.5	1,036	266,608	58.8	33.0*	−36%
Other	1,025	408,022	46.1	22.7	918	186,676	41.2	21.7	−4%

*Statistically significant at the .05 level.

Data Source: BRFSS 2003 – 2008.

**Figure 1:**
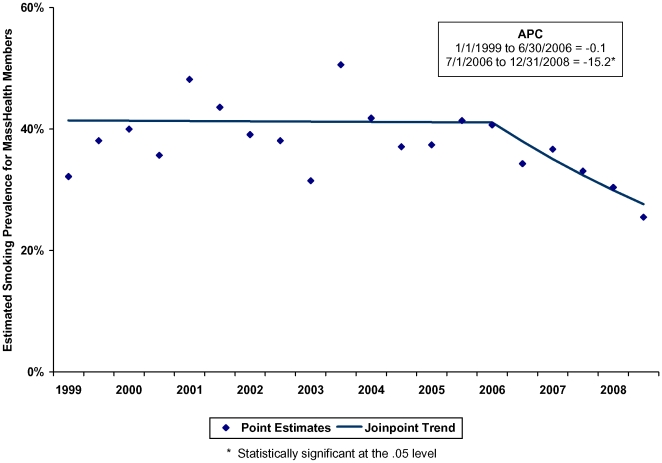
Demographic-Adjusted Smoking Prevalence of MassHealth Members, Age 18–64, 1999 to 2008 (Joinpoint Trend). (1) The diamonds on the chart represent the 6-month smoking prevalence estimates based on responses to the BRFSS. Initial weighting of prevalence estimates used a standard BRFSS weighting scheme in which data are directly weighted for the probability of selection of a telephone number, the number of adults in a household, and the number of telephones in a household. A final post-stratification adjustment is made for non-response and non-coverage of households without telephones. Data were also weighted in such a way to force prevalence estimates to match demographic characteristics for the period from 1/1/2003 through 6/30/2006. (2) The lines on the chart represent the smoking prevalence trends for the MassHealth population as estimated by the joinpoint analysis. The period between 1/1/1999 and 6/30/2006 showed no significant change (p = 0.93). Beginning 7/1/2006, there was a significant downward trend (p<0.05).

Joinpoint trend analyses were computed for smoking prevalence between 1999 and 2008. Since information about quit success was not asked in every year, trend analyses were not computed for quit success. Results showed that a model with one joinpoint was the best fit for prevalence estimates between 1999 through 2008. The sole joinpoint corresponded precisely with the implementation of the MassHealth tobacco cessation benefit (see Figure 1). Prior to July 2006, there was no significant change in smoking prevalence among the MassHealth population. Beginning in July 2006, demographically adjusted smoking prevalence dropped at an annual rate of 15.2% (see Table 3).

**Table 3:** Demographic-Adjusted Smoking Prevalence for Joinpoint Analysis, 1999–2008.

Six Month Period	Demographic-Adjusted Smoking Prevalence		Joinpoint Predicted Value
	Estimate	Standard Error	
1	32.2%	5.4%	41.42%
2	38.1%	4.7%	41.39%
3	40.0%	4.4%	41.37%
4	35.7%	4.1%	41.35%
5	48.2%	4.0%	41.33%
6	43.6%	1.8%	41.30%
7	39.1%	4.0%	41.28%
8	38.1%	4.0%	41.26%
9	31.5%	4.0%	41.24%
10	50.6%	7.7%	41.21%
11	41.8%	7.7%	41.19%
12	37.1%	5.1%	41.17%
13	37.4%	5.1%	41.15%
14	41.4%	1.6%	41.12%
15 (Joinpoint 1)	40.7%	3.7%	41.10%
16	34.3%	3.7%	37.97%
17	36.7%	3.0%	35.08%
18	33.1%	2.2%	32.40%
19	30.4%	2.4%	29.93%
20	25.5%	3.0%	27.65%

Data Source: BRFSS 1999 – 2008.

Finally, in order to make individual level adjustments for demographics, a logistic regression was computed. The target variable was current smoking as recorded by the BRFSS. Two time variables were included in this analysis: a long-term trend variable and one that would measure changes in the post-benefit period only. Demographic adjusters were also included. The long-term trend in smoking prevalence over the entire time period (1999 through 2008) was non-significant (p = 0.60). However, the trend in the post-benefit period showed a significant decrease (p<.001) with an estimated annual decrease of 15.0% per year. See Table 4 for details.

**Table 4:** Logistic Regression on Smoking Prevalence with Trend and Demographic Independent Variables, 1999–2008.

Parameter	Estimate	Odds Ratio	Pr > ChiSq
Overall Trend (monthly)	0.000582	1.001	0.60
Post-Benefit Trend (monthly)	−0.0135	0.987	0.0004
Age	−0.0128	0.987	<.0001
Gender • Male vs. Female (ref.)	0.0554	1.117	0.06
Race/ethnicity • White vs. non-White (ref.)	0.5608	3.070	<.0001
Education • < HS vs. 4+ years of college (ref.) • HS vs. 4+ years of college (ref.) • Some college vs. 4+ years (ref.)	0.51900.25000.0649	3.8682.9562.956	<.0001<.0001<.0001

Data Source: BRFSS 1999 – 2008.

## Discussion

The Massachusetts experience suggests that a good benefit design, combined with broad promotion, can result in a significant reduction in smoking prevalence. In the past 20 years, dramatic reductions occurred in smoking prevalence among the college educated in Massachusetts. These results suggest that when offered easy access to low-cost medications, the Medicaid population can also show significant reductions in smoking prevalence. Furthermore, there was a significant increase in quit success without any corresponding increase in the proportion of smokers making quit attempts. Further research is required to determine the role of promotion in the decrease in smoking prevalence in this population. Data was not available in the Massachusetts BRFSS to determine whether there was any increase in evidence-based quit attempts in the post-benefit period.

Several limitations should be noted. Smoking prevalence might be increasingly underestimated by BRFSS traditional survey method because adults lacking landlines are more likely than the general population to smoke [6]. However, systematic bias introduced by declining response rates or the ongoing trend away from landlines would have been gradual. In contrast, the joinpoint analysis and logistic regression suggest a sharp change in smoking prevalence trend. Estimates of smoking prevalence were based on self-report, but self-reported smoking status has been shown to have high validity [7].

Also, enrollment in MassHealth increased following health reform. While much of this increase may have come from the rolls of the previously uninsured, most uninsured found insurance through other programs [6]. Responses to the BRFSS did not include questions about the length of time one was insured through any particular insurer, therefore it cannot be precisely determined how much the increased enrollment affected prevalence estimates. To partially account for these demographic changes resulting from enrollment increases, prevalence estimates were computed using a weighting scheme that forced the demographic characteristics of the post-benefit period to match those in the pre-benefit period.

Finally, smoking cessation was promoted broadly to the full Massachusetts population in several ways during the study time period. For example, MTCP ran a general media campaign November 2007 – January 2008; pharmaceutical companies advertised products for cessation; and on July 1, 2008, the state excise tax increased by $1 per pack and the state quitline began offering free nicotine patches to callers. The proportion of MassHealth subscribers among quitline callers did not change between 2005 and 2008. Thus, it seems unlikely that broad-based actions such as advertising, as opposed to the tobacco cessation treatment itself, are the primary explanations for MassHealth subscribers' higher quit rate over the last 2 years.

Information comparable to that reported here for Massachusetts has not been published for other states or the U.S. as a whole. The crucial health implications of preliminary findings from Massachusetts strongly suggest that similar analyses be undertaken in other states. Variations across states in level of benefits, ease of access to services, extent of advertising and other promotion of benefits, and baseline smoking prevalence provide opportunities for comparative analyses that could help identify variables that foster the largest possible impacts of benefits. Subsequent research might focus on linking drops in smoking prevalence to improved health outcomes, reduction in claims, and specific cost-containment strategies.

The Public Health Service's Clinical Practice Guideline for treating tobacco use and dependence recommends that both medication and counseling be offered to patients [8]. Similarly, offering cessation services is an integral part of the World Health Organization's MPOWER policy package for reversing the tobacco epidemic [9]. The Massachusetts tobacco cessation benefit claims utilization data are, by inspection, suggestive that pharmacotherapy treatments might be particularly promising in terms of probability of being utilized. One possible reason why cessation counseling was little used by MassHealth subscribers is that relatively few primary care settings had the staff resources needed to make 30- or 60-minute tobacco treatment sessions readily available. Although speculative, it seems likely that many office encounters leading to prescriptions for tobacco cessation medications also included caregiver discussion and advice on quitting, even if counseling was not the primary purpose of the visit.

The Massachusetts findings suggest that a broadly-promoted, accessible, comprehensive smoking cessation benefit can reduce smoking prevalence in the Medicaid population. In 2004, U.S. Medicaid expenditures for smoking-related conditions totaled $22 billion [10]. Tobacco cessation treatment is cost-effective and should be made available to all smokers [11] via health insurance benefits. Fully implementing known tobacco control strategies has strong promise to end the U.S. tobacco epidemic [12].
